# Enabling Spaces; Rethinking Materiality and the Invitational Character of Institutional Environments

**DOI:** 10.3390/ijerph19095577

**Published:** 2022-05-04

**Authors:** Emma Nielsen, Sofie Pedersen

**Affiliations:** 1 Carlberg Aps, 2450 Copenhagen, Denmark; 2 Social Psychology of Everyday Life, Department of People & Technology, Roskilde University, 4000 Roskilde, Denmark

**Keywords:** enabling spaces, liminality, liminal hotspots, behavior settings, affordances, universal design, inclusive design, social work, lived citizenship

## Abstract

This article explores how physical surroundings may be integrated as a supportive measure in social work efforts. Drawing on ecological psychology and the concept of liminality, the article presents a case study of Kofoed’s School (KS), a social institution in Copenhagen, Denmark. In recent years, KS has undergone a major renovation, opening up previously sheltered workshops to the public. By creating liminal spaces of possibility, where students can take up “both/and” positions allowing for a multitude of ways to participate, students are experiencing increased support and inclusion, which contributes to a growing feeling of citizenship and well-being. Drawing on participant observations and interviews with students, staff members, as well as customers at the school’s shops, we explore how the architectural layout may facilitate students’ flexible and fluid movements between more or less sheltered positions and further discuss how this flexibility may become supportive for their personal development and well-being. We propose to think of such spaces of possibility as enabling spaces, where inclusive architecture contributes to the creation of new possibilities for participation for people in marginalized life positions. This, we suggest, holds a great potential for social work efforts for people experiencing complex social vulnerability.

## 1. Introduction

Social work is a complex field of interacting aspects, where some are given more priority than others in our attempts at understanding the effect of various interventions. Most often, it seems that formal conditions, such as political directives and legal restrictions, are emphasized along with pedagogical approaches. These are, unquestionably, aspects of utmost importance when trying to best facilitate and support individual development, rehabilitation, or resocialization. However, another—and more informal—aspect is rarely included in our analysis of what contributes to positive outcomes of social work; namely the significance of the physical environment, e.g., the architectural layout of the institutional setting in which the social work effort is carried out. The material or physical conditions tend to become a black box about which we know hardly anything, nor is it something we care to pay attention to. This is especially true when our social work efforts extend to socially marginalized people [1]. Hence, this opens a research question of *how the physical surroundings play a part in the (developmental, social) processes that occur in social work settings*, and—perhaps more importantly—*what are the implications of this for practice?*

To further explore this question, the article will present a case study of Kofoed’s School (KS), a social work setting in Copenhagen offering sheltered workshops for people in marginalized positions. KS has in recent years undergone a major renovation of the school’s physical layout which has created new possibilities for action for the students at the school, and furthermore facilitated a larger degree of integration within the local community. Drawing on participant observations and interviews with students, staff members, as well as customers at the school’s shops, the article explores how the architectural layout may facilitate students’ flexible and fluid movements between more or less sheltered positions and further discusses how this flexibility may become supportive for their personal development and well-being. We shall argue that by considering the architectural layout a more integral part of social work efforts, we may enrich the ways in which we can support the participation and well-being of people in marginalized societal positions.

Before we introduce Kofoed’s School more concretely, we shall briefly (1) position the article in relation to general movements towards a more inclusive architecture, and (2) consider the meaning of creating ‘enabling spaces’ for participation.

### 1.1. Towards an Inclusive Architecture

Over the last 50 years, disability activists, designers, and social scientists have called for a need to rethink how we design our physical spaces to create more accessible and inclusive environments [2,3]. In 1971, designer Victor Papenek argued that design was failing to accommodate non-normative bodies. He argued that we all at some point will experience having what could be described as ‘special needs’, and when designers fail to design for e.g., older people, people with disabilities, children, or anybody who falls outside the statistic average, they no longer design for the majority, but for a minority [2,4]. Grassroots movements such as the CRIP- and ‘Barrier Free’-movements similarly protested the lack of accessible design, and in 1985 disabled architect Ronald Mace introduced the term Universal Design, which has been used as both a theoretical approach and methodology within industrial design and architecture [5]. Mace describes Universal Design as: “... the design of products and environments to be usable by all people, to the greatest extent possible, without the need for adaptation or specialized design” [6].

The philosophy of Universal Design has since been included in the UN’s convention of the Rights of Persons with Disabilities [5,7] and has led to a variety of guidelines, goals, and standards for how to create more accessible spaces and design (see e.g., Steinfeld et al. [8] and the EIDD Stockholm Declaration [9]). Most recently, in 2020, the European Commission approved a European-wide standard for a "built environment accessible for all", drawing on Universal Design [10,11]. In our opinion, these were important steps towards creating a more inclusive built environment.

However, Universal Design has been critiqued for mainly focusing on physical impairment, and too much on creating ‘one-size-fits-all’-solutions. [12,13]. As a result, the approach has evolved into terms such as ‘Inclusive Design’ or ‘Inclusive Architecture’, which point beyond the emphasis on people with disabilities [12]. By drawing on a more intersectional approach, Inclusive Design also focuses on mechanisms of exclusion caused by e.g., mental health and psychosocial issues, social and economic marginalization, age, race, gender, and culture [2,5]. Compared to the Universal Approach, Inclusive Design instead examines how we can create a *multiplicity of solutions*, which allows more people to be included [12,14].

Despite a growing interest in Universal Design and Inclusive Design, Denmark is falling behind in terms of incorporating this understanding in legislation, compared to, for example, Norway, where Universal Design is included in state legislation under the headline ‘Universell Utformning’ [5,12]. The Danish Building Regulations do include policies about accessibility, but primarily in the sense of accessibility for those who are visually impaired or who have difficulty walking (e.g., requiring wheelchair access). Hence, they have yet to incorporate how the built environment may help overcome other types of exclusion, e.g., as experienced by people facing mental health issues, psychosocial problems, or marginalization. 

Furthermore, according to Grangaard, most developers stick to the regulations rather than push for a more inclusive design, as they perceive it as extra costs, rather than an opportunity for adding value [15]. A general lack of inspiring examples, economic calculations, and follow-up evaluations mean that developers, architects, and legislators are unaware of the opportunities and concrete ways in which they can incorporate efforts to develop more inclusive architecture [15] (p. 6). 

However, within recent years, some of the largest Danish foundations (A.P. Møller Foundation, Realdania, Bevica Foundation) have started working with Universal Design and Inclusive Architecture under the headline ‘Social Bricks’, focusing on the interplay of social institutions and people experiencing complex social marginalization. This has led to funding the development of new buildings, evaluations of existing architecture, as well as research within the area (See e.g., Realdania [16] and the A.P. Møller Foundation [17]). The renovation of Kofoed’s School was part of the ‘Social Bricks’ initiative, with a primary aim of understanding and developing a physical setting to support both social work efforts and the individual needs and life experiences of the students at the school.

Although academics, activists, designers, and foundations have worked with universal and inclusive design, aiming to create more inclusive places for people with various physical and mental disabilities, a long way remains for it to be included more widely in practice and legislation. One of the main impediments is a lack of research as well as relevant case examples [1].

### 1.2. Creating ‘Enabling Spaces’ for Recovery

The awareness brought on by Universal- and Inclusive Design can, in our opinion, contribute to the creation of what we would label *enabling spaces*: spaces where new forms of (social) participation is invited for and enabled for people in societally marginalized positions. When addressing complex psychosocial problems, such as mental health issues, homelessness, drug abuse problems etc., problem conceptualization as well as intervention is often directed at the individual. However, as Davidson states: “*Citizenship and participation in society are no longer considered the gain from recovery*, *but rather the prerequisite for even being in recovery*” [18] (p. 35, our translation from Danish). Furthermore, Topor, Larsen & Bøe [19] propose to redefine our comprehension of recovery to include a profoundly social foundation. In their revision of the widely used definition by Bill Anthony from 1993, they write:

Recovery is a deeply **social,** unique and **shared process** in which our living conditions, **material surroundings**, attitudes, values, feelings, skills, and/or roles are changing. It is a way of living satisfying, hopeful, and reciprocal lives, together with others even though we may still experience distress, unusual experiences and troubled or troubling behaviour. Recovery involves **engaging in new material and social contexts** and in open dialogues where new ways of understanding and handling the situation are created as we move beyond the psycho-social-material crisis (emphasis added).

Furthermore, they state how recovery-oriented work is not about definitions alone, but rather about addressing societal challenges and providing social work efforts that seek to “diminish inequalities when it comes to economy, housing, schools, and local environment”. This resonates with how the notion of citizenship and equal rights have been an integral part of Universal Design from the onset. In this perspective, citizenship is not only something that happens in the official relation between state and citizen (as when a person is allowed citizenship), but rather something that is enacted and negotiated every day in the realm of the quotidian relations and interactions with our surroundings—also referred to as ‘lived’ or ‘everyday citizenship’ [20,21,22].

Therefore, in this paper, we are interested in the possibilities for action that arise in the intersection of (1) the physical surroundings, and (2) how people make use of the spaces in ways that support social participation and citizenship as a basis for recovery in a broad sense. The architectural design and concrete use of spaces in social work settings may, we suspect, readily support the pedagogical approaches aimed at promoting citizenship and social inclusion, both of which are important aspects of general well-being and social sustainability. 

In the renovation at KS, user involvement and the specific needs of the users has guided the design process. In that sense, it can be regarded in a Universal Design perspective, and it makes the school an interesting case for the exploration of the meaning of the person-environment relation in social work.

Before we begin our case analysis, we shall provide an introductory framing of Kofoed’s School, present the theoretical framing of the article as well as account for the methodological approach and empirical material. The analysis is structured around the exploration of two different focus points that in their own way open up to reflections about the relationship between the concrete environment and people’s action possibilities. The analysis will be followed by a discussion and a conclusion.

## 2. Introduction to Kofoed’s School (KS)

KS was founded in 1928 to support the many unemployed young men in Copenhagen at the time. Since then, the school has become one of the city’s biggest actors in terms of supporting people out of employment or who are otherwise experiencing marginalization, such as homelessness, substance abuse, and/or mental or somatic health issues. [23]. Today, around 3500 people are signed up to attend either short courses or to take part in the various workshops that KS offers. As the users of KS are called “students”, we shall refer to them as such henceforth.

**Scheme 1:**
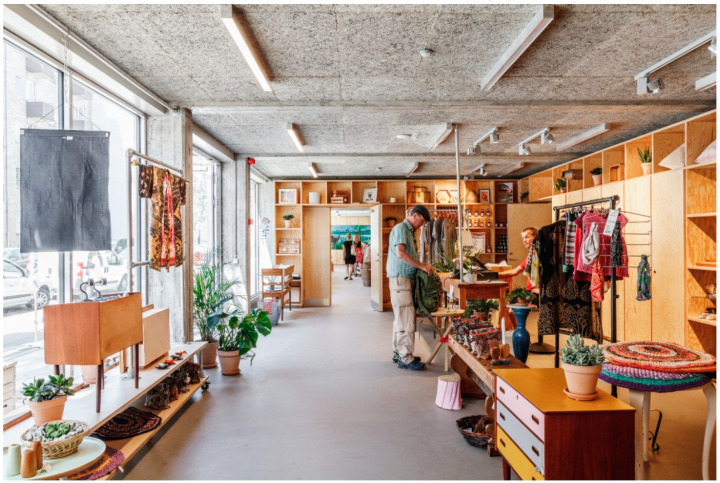
The Bazaar, Photo: R. Hjortshøj for Kofoeds Skole.

**Figure 1:**
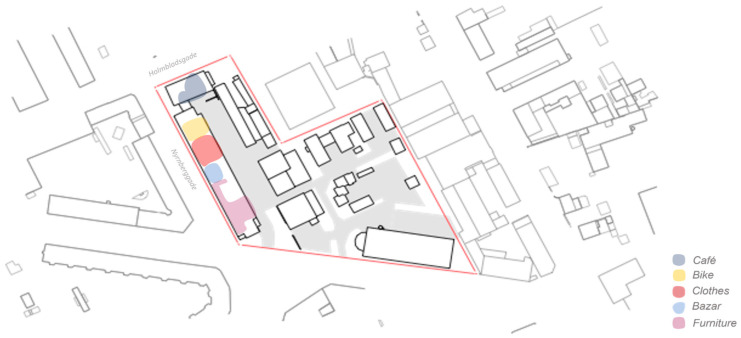
Map of KS area.

**Figure 2:**
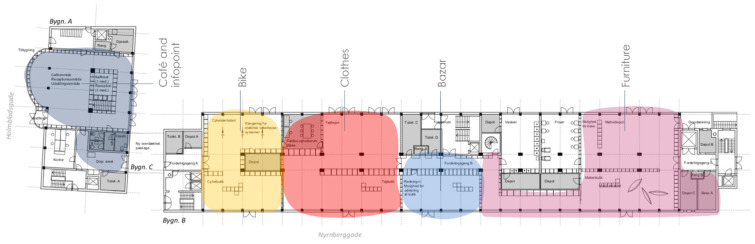
Floor plan of the ground floor at KS with the four social economic shops and café.

Between 2014–2018, KS underwent a major renovation which has had a significant impact on the school’s pedagogical practice. The renovation had many goals, some of which included enhancing the school’s economy, creating a greater connection between the students and the surrounding neighborhood (by opening a range of social shops and a community café), upgrading the buildings and making them more sustainable for the future, and finally, creating supportive learning environments. These were based on the user involvement of both the school’s students and staff. In their refurbishment, KS has worked actively with the creation of new material settings and opportunities, such as by transforming the ground floor into a café and four new shops: a bike shop, a vintage clothing shop, a secondhand furniture shop, and a “bazaar” [24,25]. The bazaar sells the arts, crafts, and delicacies produced in the school’s other workshops, such as homemade apple juice and honey, homemade Greenland mittens, hand knitted scarves, and hand-crafted wood furniture (see Scheme 1). Figure 1 and Figure 2 (below) show a map of the KS area and the ground floor plan with color indications of the different workshop areas.

The design principles and process behind the renovation will be further elaborated in the analysis.

## 3. Theoretical Framing

The theoretical framework of this paper draws on different perspectives that, in combination, allows for the analysis of what we will propose as *enabling spaces* in the intersection of people and the built environment. In the following section we will introduce key concepts and understandings from ecological psychology to understand how physical surroundings may support the pedagogical practices as well as the participation of those who inhabit these surroundings. From here, we will introduce the notion of liminality to further explore how the physical layout can create not only *inclusive architecture* but become *enabling spaces* when liminal positions are taken into account and designed for.

### 3.1. Ecological Psychology: How the Built Environment Affords Certain Actions

To analyze the meaning of the built environment in relation to the space of possibilities created at KS, we need a theoretical comprehension of the invitational character of the environment. Here we draw on James Gibson [26] and his notion of affordances: affordances account for the complementarity of the person and the environment, and thus allows for an understanding of the way in which the environment *invites* certain actions. Peoples’ actions are not just reactions to stimuli, nor are they results of mental processes alone. Rather, we need to comprehend people’s actions as the result of a person-environment reciprocity, putting the functional possibilities of the environment into focus [27]. 

The invitational character of the environment is further nuanced by Roger Barker and Herbert Wright [28], who proposed the term *behavior setting*. Their extensive analyses of life in the small town of Midwest from the late 1940s and onwards have contributed significantly to the understanding of the environment in relation to behavior and behavioral opportunities. Barker and Wright pointed out how behavior settings, meaning the properties and availabilities of the concrete situational environment, are in fact stronger predictors of behavior than the individual personality. As Wright et al. wrote:

Behavior settings are coercive. Every adult who has yelled at a ball game, bowed his head at church, ridden all day on a train, or listened at a concert knows this to be true. So does every child who has sat tight in his assigned seat in a classroom. With frequent and important exceptions, in any behavior setting different persons do like things in similar ways. It has to be recognized, however, that there is no binding dynamical relationship between behavior setting and human action. In any setting anything can happen—as a teacher facing a classroom full of children knows well. [...] The coercive effect of a setting upon behavior, then, is indirect. It stems only from the fact that every setting tends to bring about certain psychological habitats rather than others.

[29] (pp. 189–190)

Without disregarding individual agency, Barker & Wright emphasized the meaning and significance of the environment in relation to how people orient themselves, and what actions they engage in. A behavior setting can thus be defined as a setting in which standardized behavior patterns tend to occur, or as “community areas which individuals can enter and in which they behave in accordance with forces that produce the characteristic behavior pattern” [28] (p. 458). A behavior setting is directly perceivable but need not refer to fixed structures in the environment exclusively, such as specific rooms or buildings. They can also appear as momentary settings, e.g., when rooms are used for specific purposes at certain times, or as recurring traditions, such as Halloween [28,30]. Furthermore, behavior settings can be further operationalized in subsettings, meaning settings within settings. 

Over the years, Barker, Wright, and colleagues conducted extensive studies of e.g., schools as behavior settings, and made comparisons between small and big schools, in relation to students’ participation and how variations in participation correlated with variations in school size (see e.g., [28,31,32]). Their findings suggested that the smaller schools invited pupils to take part in considerably more behavior settings than did larger schools, which suggests not only a difference in the meaning of school size in relation to behavior, but also that behavior settings must be explored concretely to comprehend their influence on participation and individual action possibilities. Hence, behavior settings are not to be comprehended as determining people’s actions in a one-to-one manner, but rather as an indicator of the meaning of the environment in relation to possibilities for action or participation.

Both Gibson’s notion of affordances as well as Barker and Wright’s notion of behavior setting can be criticized for not thoroughly integrating the subjective or historical aspects of the human lifeworld (see [29,33]). We are not blind to such criticisms, nor do we disregard individual agency as meaningful. However, for present purposes, we will take our starting points in these conceptual awarenesses, as presented by Gibson and Barker and Wright, in order to magnify the meaning of the physical properties of the environment, not at the expense of individual agency, but as a way to emphasize and explore the meaning of that which is often omitted in our comprehension of social work. 

### 3.2. Liminality as Concurrent Challenge and Possibility

To supplement the ecological perspective, we will draw on the notion of liminality (acc. Turner [34,35]), and the possibilities inherent in liminal hotspots (as proposed by Greco & Stenner [36]; Stenner, Greco & Motzkau [37]). In his work on liminality, Turner [34,35] draws on the anthropologist Arnold van Gennep’s work on rites of passage to mark the ‘space between’ more established societal categories, such as ‘child’ and ‘adult’. The transition between such categories were often associated with various rites of passage, as the Catholic confirmation, or graduating school. Transitional periods in people’s lives often involve a certain uncertainty and ambiguity concerning self-understanding, capabilities, and even meaning and direction, or between ‘the having been and the becoming’ [34] (p. 234). Such transitional times can be conceptualized as *liminal*; a being in between societal positions or categories—en route, but not yet there.

The liminal position or space is considered challenging due to its inbuilt ambiguous nature; however it is also a space of possibilities, in the sense that newness may (and is expected to) arise and take form, new doors may open, and new capabilities may be developed and manifested. In their further development of Turner’s concept, Greco and Stenner propose the term ‘liminal hotspot’ to refer to the “experience of being trapped in the interstitial dimension between different forms-of-process, and in the situation of ontological indeterminacy that characterizes such a dimension” [36] (p. 152). As Stenner, Greco & Motzkau further wrote: “The liminal experiences of ambiguity and uncertainty that are typically at play in transitional circumstances acquire an enduring quality that can be described as a “hotspot”. Liminal hotspots are characterized by dynamics of paradox, paralysis, and polarization, but they also intensify the potential of pattern shift” [37] (p. 141). It is exactly this inherent intensified potential of pattern shift or of the co-existence of that which is possible and impossible (the paradoxical) in relation to participation—and ultimately inclusion/exclusion—that we wish to explore.

By employing a notion of liminality, we hope to be able to nuance our comprehension of the ways in which *new spaces of possibilities* are created for the students at Kofoed’s School by enabling various modes of being in the room: e.g., as fully taking part (acquiring a work identity), as active on the sideline, as observing, or as being outside (not able to take part in the activities at KS, or needing a time-out (e.g., due to social anxiety)). Not as fixed and mutually excluding categories, but more so as fluid and dynamic positions that can change according to individual needs. These various modes of being are not normative in the sense that all users are expected to move towards a ‘full participation’ in a uniform sense, or as a linear movement, but rather that the possibility of *being able to move freely* between such different modes of being in itself allows users to move with their emotionality and hence experience a sense of mastery. Hence, liminality comes to designate a multiplicity of modes of being, which, as we see it, corresponds with the manifold microgenetic developmental movements that students at KS do. By shedding light on the interplay of the built environment and how people make use of the space, participate, and see themselves in this setting, we may further nuance our comprehension of what works in such social work efforts—and how the concrete environment has a constitutive role to play.

## 4. Methodology and Empirical Material

In this paper, we take an interdisciplinary approach linking psychology and architecture. The case study draws upon data gathered during fieldwork conducted at Kofoed’s School using a qualitative research approach combining participant observations with semi-structured interviews and more informal conversations with staff, students, volunteers, and community representatives as well as neighbors and customers in the shops.

Participant observations were conducted over three weeks in October 2019 by one of the authors (E. Nielsen) and carried out on the ground floor of KS, which hosts the school’s four workshops and the café. Participant observations were chosen for several reasons: (1) as a means to understand *how it feels* to participate in the selected workshops and to acquire a personal (bodily) experience of how the concrete architectural layouts of the respective settings afford certain actions and experiences rather than others [26,38]; and (2) as a way to have conversations (and informal interviews) with the students *during* activities, where the concrete environment or activities were used as probes to understand *how they experience* the new physical settings [38]. And finally, (3) as the students are often vulnerable and do not necessarily have good experiences from past interview situations or conversations with authorities, the school recommended an approach where we engaged with students during *shared activities.* As an ethical consideration, students were informed of the aim of the research and given the opportunity to opt out at any point, should they not want to participate. It was important to us that students did not feel any pressure to disclose information that they were not comfortable with, just as it was important to convey that we were not interested in the personal histories or trajectories of individual students, but rather in their experience and use of the physical environment and what the concrete surroundings meant for their participation. Fieldnotes were taken concurrently with observations [39] and interviews.

During the three weeks of field work, a total of 26 interviews were conducted, ranging from semi-structured interviews to more informal conversations with staff (10), students and volunteers (16). Whereas the participant observations may inform us about *the use* of the workshops (as new behavior settings), the informal and semi-structured interviews were chosen as a means to understand students’ and staff’s *experiences* and *perceptions* of the new physical layout [38,40]. The interviews followed a qualitative, semi-structured approach with questions focusing on the thoughts of students and staff regarding the renovation (of the school’s ground floor premises), how they experienced the new space(s), what they were able to do now compared to before, and what they thought it meant for how the school was perceived by the outside world. These data were supplemented with four interviews with community representatives from the surrounding neighborhood as well as 24 shorter interviews or conversations with customers in the shops. These interviews and conversations were conducted to get a better understanding of how the redesign of the institutional space (both visibly and functionally with the social enterprises) might have impacted the general public’s understanding of the school and its users.

The qualitative approach proved well-suited for an in-depth study of the relation between the physical settings and the behavior of the students and staff. When novel views or knowledge emerged from either interviews or participant observations, these were tested or used as probes to further explore how the physical setting afforded certain behaviors and experiences amongst students, staff, and guests/customers, and to test whether this represented isolated opinions and experiences, or something more general. Hence, the data excerpts that are included in the article have been selected as they represent more general experiences (that appeared across several participants), and so provide insight into more general impressions or tendencies in the empirical material. This does not imply that they can be generalized in a one-to-one manner to other similar settings; however, by exploring KS rather concretely (combining several qualitative approaches), we acquire situated knowledge about how person-environment exchanges may unfold in a particular social work setting. This provides us with concrete impressions of how the physical surroundings play a part in the social dynamics that unfold at the school, and of the difference that the architectural redesign has made in relation to students’ individual developments (in terms of how they participate and see themselves). Our hope is that this will serve as inspiration for other similar practices, or for new questions to be asked, when (1) considering the design or redesign of social work enterprises, and (2) when trying to comprehend how people act and experience themselves in such settings.

As the research was carried out in collaboration with Kofoed’s School, the school appears by its real name in this article. However, the names of participants in the project (users, staff, customers) have been anonymized for ethical reasons. 

The analytical strategy employed in this article consists of co-exploring the new architectural layout and students’ experiences of these new settings, with an emphasis on how the different settings come to serve as enabling spaces for students’ participation. Focus points as well as data excerpts have been selected accordingly to explore variations and the (architectural) support of new possibilities for action. Hence the analysis will solely explore the settings of Clothes and Furniture, respectively, as these are emblematic of how the architectural choices and solutions may work to support social work efforts at KS. 

Throughout the case analysis, we will use capital letters when referring to specific areas at KS (Clothes, Furniture) or to the design principles that were employed (School, Base, City). We will also be using architectural drawings (floor plans) of the various locations, as well as photos from the school, to support the analysis. These appear by permission.

## 5. Case Analysis

We will begin our analysis by looking at the design principles employed at the school and visually present a model of the ground floor to support our analytical exploration. From there on, we will look at how the behavior settings have changed with the renovation and use this as a vantage point for further exploring different aspects of the person-environment interplay. 

### 5.1. Meaningful Design Principles: Base-School-City

An important feature of the refurbishment of the ground floor was a set of design principles developed in collaboration between the school, the architecture company Cobe, and consultants Carlberg|Christensen. The overarching design principle was to create different zones to ensure that the new openness (with the public-facing shops and café) was countered with more sheltered areas. The purpose was to allow for users to participate in more or less publicly exposed ways, according to their needs and moods, and to alternate between different ‘levels’ of exposure more freely and flexibly [24]. This design principle was named ‘Base-School-City’ [24]: ‘Base’ was used to describe the most sheltered areas, with keywords being homeliness, contemplation, and security; ‘School’ describes areas containing the ‘school’-activities that KS offers, such as workspaces and learning environments with a social pedagogical approach based on help-to-self-help principles; and finally, ‘City’ describes areas with a large degree of openness and exchange with the surrounding city (‘the outside world’) [25] (p. 11). Here the borders between school and non-school are permeated and fluid. 

**Figure 3:**
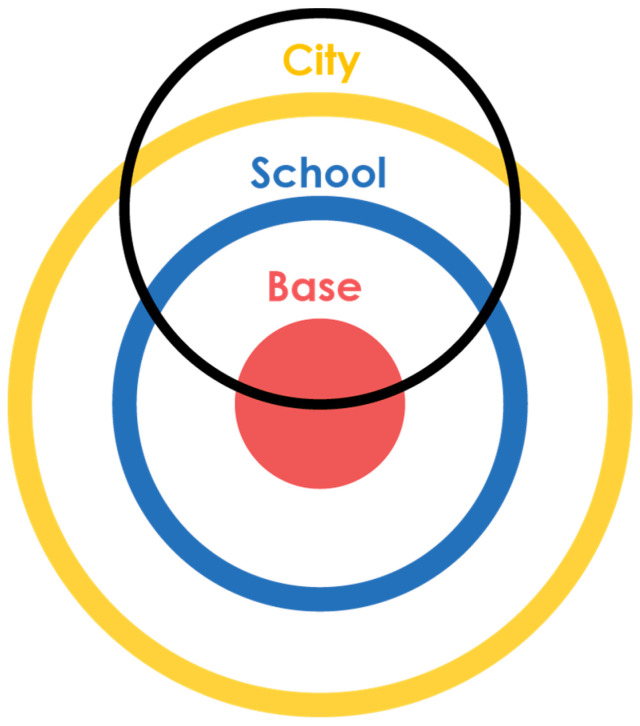
The design principle has created a spatial span between a more sheltered Base-zone and the public facing City-zone [25] (p. 11). The different dimensions are represented both in the overall layout of the school, from the public facing facade to the more sheltered garden and upper floors, as well as within the individual workshops, where all three zones have been included.

**Figure 4:**
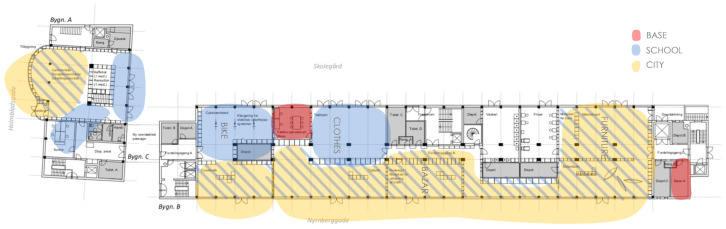
Layout of the ground floor at Kofoed’s School. The red markings indicate the Base areas, with room for social withdrawal and privacy. The blue markings indicate the School areas with emphasis on workshop and learning spaces, and the yellow markings indicate the City areas, where boundaries between the school and the public are fluid and flexible.

The principle described as “Base-School-City” thus defines three zones ranging from sheltered, intimate space (the base) to a space open to the public (the city). See Figure 3 and Figure 4.

The early stages of the refurbishment process included an extensive involvement of both staff and students, which informed the key design principles. A shared main concern was that KS would no longer be experienced as a safe space and refuge for the students [24] (p. 29). Where the initial focus of the refurbishment had focused on making the school more open and accessible to the public, the engagement of students informed the need for more sheltered areas, and so the principle of ‘Base’ was developed. Sheltered areas were deemed important for students to be able to retire or take breaks if needed; either due to mental fatigue or (social) overexposure. 

Such awareness shows the importance of involving users from the early stages of the design process to ensure that the spaces created are deemed relevant and meaningful for them, which resonates with the principles of the Universal Design and Inclusive Architecture approaches [5,8]. It demonstrates how good design in social institutions is not just a question of aesthetics or the best usage of space (as optimization), but rather the result of a close collaboration between designers, users, and professionals in the field. The creation of meaningful, inclusive spaces requires in-depth understanding of the user group and the purpose of the institutional setting, and thus central questions become: what is to be promoted and facilitated here, and which challenges is the institutional setting targeted at? This is perhaps even more important when designing spaces for people who are dealing with various mental or psychosocial issues, and for whom participation outside their home setting may be strenuous and demanding. The user group at Kofoed’s School may also have experiences of prior participation in a variety of institutional settings in which they have not felt welcome, normal, safe, or supported. 

The design principles at Kofoed’s School have had a significant impact not only on the way in which the space and architecture has been designed, but also on the pedagogical approach and the way in which the school works, including students’ experiences and the public’s view of the school, which will be explored further in the following section.

### 5.2. Not Just a School—The Introduction of a New Behavior Setting 

Traditionally, social institutions like Kofoed’s School have offered support and care through sheltered workshops by providing a space to learn new skills, keep active, have something to wake up to, as well as a safe space in which to participate. A space that allows for feeling vulnerable or unable to perform, and yet still makes you feel welcome.

For the students at KS, being part of a daily routine and a work environment can seem like an invincible task due to a variety of mental health issues, substance abuse problems, general marginalization, or perhaps homelessness. Moving from a life situation with little to no expectations of more formalized and consistent participation into a space, where expectations from colleagues may be experienced as pressure to perform may be no easy task for students, and yet, this kind of consistent participation is what many desire. To manage this liminal position, of feeling in-between an identity of being unemployed and one of having a work identity, students employ a variety of strategies to try to meet new demands, all the while feeling vulnerable and in need of extra care, time, and support. 

**Scheme 2:**
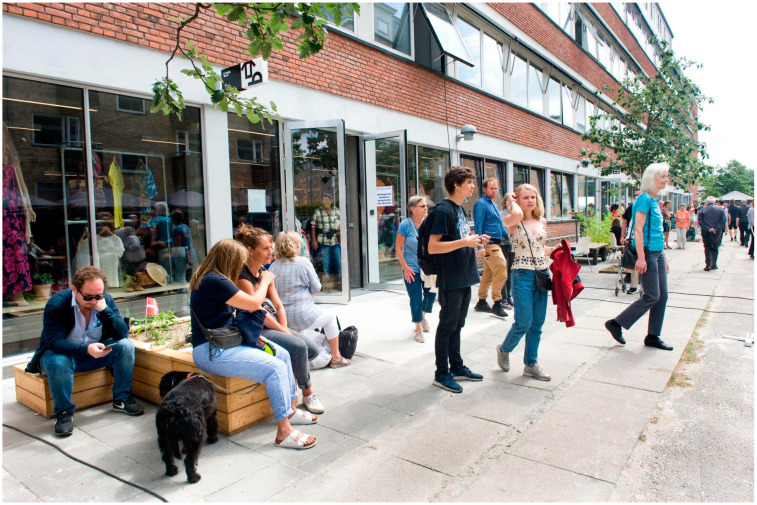
The opening day of the shops, Photo: L. Tuxen for Realdania.

With the redesign of the school to a more open and inclusive architectural layout, however, new behavior settings were created, affording new possible positions and activities in the different behavior settings at KS. Now, it is not only a safe space for the students but also a place for the public to enter, shop, and browse the clothes racks and the furniture exhibitions (see Scheme 2). Hence, the public may ‘transcend the borders’ of the institution in new ways, creating new and multiple expectations for ‘how to be’ in the rooms, e.g., in the different (work)shops. Still, the shops need to function as learning environments and safe spaces for the students, but now, as neighbors enter to shop, they also become public spaces, which may challenge students’ experience of a haven or safe space when they feel vulnerable and in need of sheltering. 

The new double function in the (work)shops in serving as both safe learning spaces as well as publicly accessible spaces, risks creating what Greco & Stenner has termed *a liminal hotspot*—a position where two types of positions or ‘forms-of-demands’ overlap spatially and temporally and “interfere with one another and you find yourself caught up in the noise between the different demands” [36] (p. 154). However, at KS, with the design of the physical surroundings, the school has managed to create a support-system and ease in the transition through the experience of liminal hotspots. This means that the liminal hotspot situation is transcended in the sense that the doubleness in the invitational character is used pedagogically by supporting a multitude and ever-changing mode of participation for the users. In the following sections of the analysis, we will explore and discuss two such ways in which the physical layout can contribute to the creation of ‘enabling spaces’, through the experience of liminality, and thus enable a greater sense of inclusion and citizenship: *The relation between School and Base* (Section 5.2.1), and *The relation between School and City* (Section 5.2.2). As noted in the Methodology section, we shall focus solely on the behavior settings of Clothes and Furniture, as these are exemplary in relation to the new possibilities for action as reflected by the refurbishment of KS, but also demonstrate the variations that exist in practice, and this may come to matter to students.

#### 5.2.1. The Relation between ‘Spaces of Work’ and ‘Spaces of Non-Work’ (the School—Base Relation) 

The two workshops of Clothes and Furniture provide two interesting and very different cases for how the built environment has become an enabling space. The workshops are in many ways similar in that they are centered around the shops: the social enterprises where recycled clothes or furniture are sold. However, the layout of the workspaces, where students help prepare either furniture or clothes for sale, as well as the Base, where students can take a break, afford rather different work experiences and opportunities to take a break in the two workshops. We shall now explore them in turn.

##### Clothes

**Figure 5:**
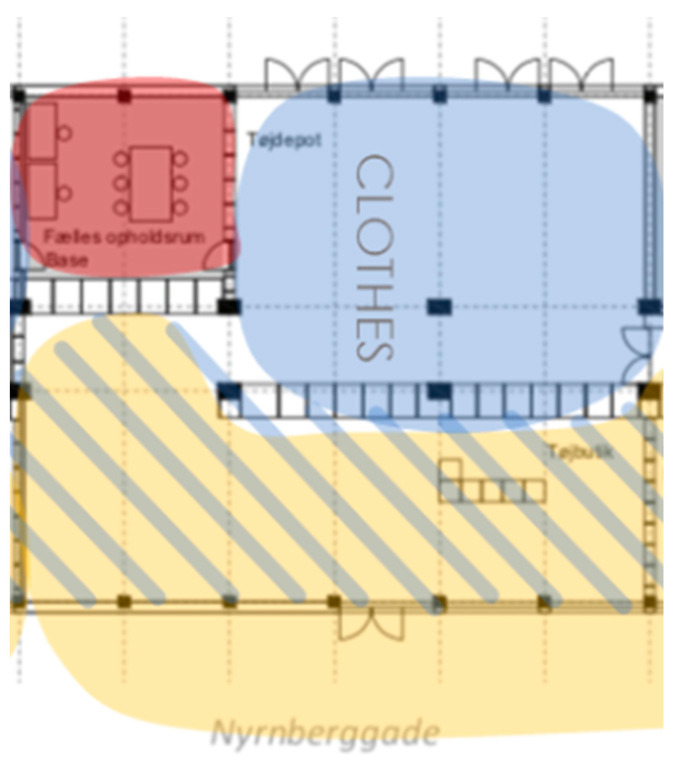
Clothes’ floor plan. Yellow area indicates shop (City), the blue area indicates workspace (School), and the red area indicates a breakroom (Base).

**Scheme 3:**
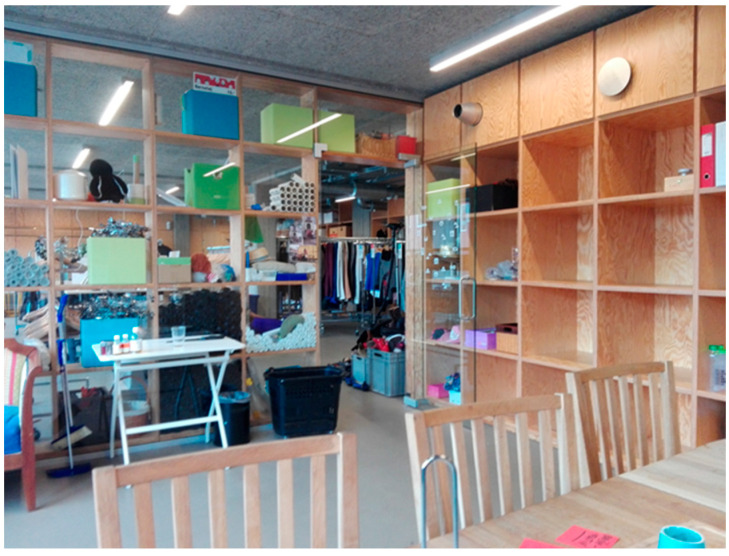
The ‘transparent’ wall between the Base in Clothes and the workspace (left).

In the Clothes section, there is a clear distinction between the three zones and what activities the zones afford. The shop-area (City) is spatially separated from the workspace (School), just as the workspace is separated from the Base (see Figure 5). However, despite the spatial separation of the three zones, they are still visually connected. No doors block the sight and the walls separating the rooms are semi-transparent and made from bookcases (see Scheme 3). 

**Scheme 4:**
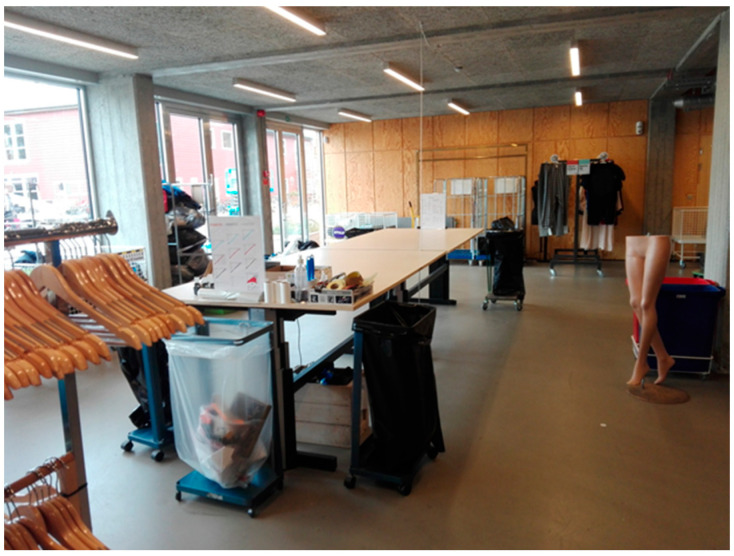
The workspace with the large central table (right). Photos by Emma Nielsen.

The workspace is centered around a large communal table, where most of the work is done (see Scheme 4). This allows students, volunteers, and staff to have conversations while working and has proved efficient for person-to-person training, including both staff-to-students and student-to-student interaction. The table thus affords a highly social atmosphere and has turned Clothes into a social hub where even students, who are not part of this workshop, pass by to have a chat and see what is going on. Here, the Base serves as a joint meeting point in the morning and provides a break opportunity during the day when someone has the need to step aside. The visual permeability of the room means that one can withdraw to the Base without feeling excluded from the sociality of the workspace—one may remain connected. One of the students, Erik, described how entering Clothes feels like being “wrapped in a warm blanket”, and another student (Marc), who was also part of Clothes at its previous location, explains how he much prefers it now:

“It is much nicer to be here because it is airier and lighter. It is more spacious, and if everything gets a bit too much, it is easy to just sneak next door and take a break”. That is the benefit of the Base he thinks. “With it (Base) being right there, it is easy to move next door if you need to talk, take a break, or get some coffee.” Conversation with Marc, Clothes.

Despite the sometimes-hectic atmosphere at Clothes due to the amount of people passing through, the students generally feel at ease here. Observations from this behavior setting point to the importance of having an integrated Base area, where one can sneak in without being excluded from social life and, furthermore, to the meaning of having a clear center in the workspace (here, the communal table), where students and staff can help each other and easily strike up conversations.

It is as if the visual connection between the Base and the workspace, and between the workspace and the shop, helps bridge the gaps between the different self-understandings afforded by the subsettings of Clothes. Hence, Clothes as a behavior setting concurrently invites students to consider themselves *students* who are learning new ways of participating and new skills in a supportive setting, as well as *employees*, who carry out necessary work tasks related to the various activities of Clothes. The fact that Clothes contains several subsettings within the same room—with different affordances—enables students to more flexibly alternate between different modes of participation, depending on how they feel. That students may withdraw from social exposure without leaving Clothes as a behavior setting was by several students experienced as supportive in relation to developing their sense of agency. The following example by John is illustrative of this:

*We met John, a student, in the Base area of Clothes. He had had a shift in the shop, where he was behind the till interacting with customers. At some point he got annoyed at a customer and withdrew to the Base area. Having this place to retreat to enabled him to relax and took the edge of things so that he could go back into the shop and continue his shift.*

In this situation, John is able to act on his emotions and care for himself by withdrawing to the Base area, where he can sit down and have a cup of coffee. However, as Base is a subsetting of Clothes and only partially secluded, he can care for himself without leaving the setting altogether (as he would have had to before the refurbishment, when Clothes did not contain this functionality). Having to leave the setting entirely would have meant a sharper demarcation line between participating and not-participating that, for John, would make it more difficult to participate, as it would feel riskier to him, or he would feel more exposed. Having the possibility to move more freely between the subsettings, without feeling that he ‘leaves’, means that John can sustain a more consistent participation which gradually helps build his feeling of agency and mastery of the situation. By observing how John participates and manages to meaningfully connect with the different possibilities for action afforded by the subsettings of Clothes, we see how the new architectural layout is experienced as supportive, and how it contributes to develop John’s sense of mastery of new skills and otherwise challenging situations.

##### Furniture

**Figure 6:**
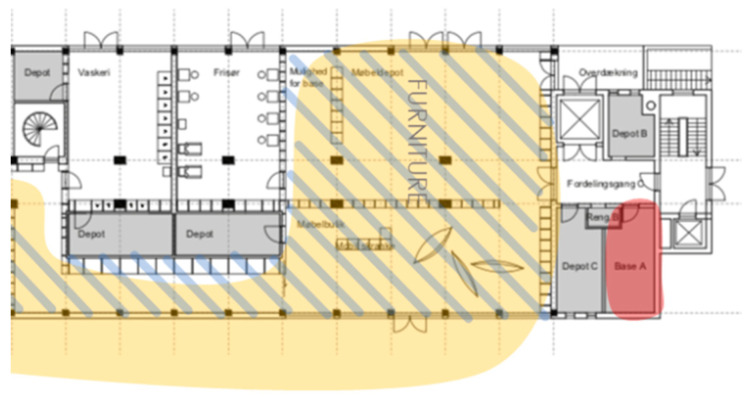
Furnitures’ floor plan. The yellow area indicates the shop (City) and the red area indicates the break-room (Base). Here the workspace area is included in the publicly accessible area.

**Scheme 5:**
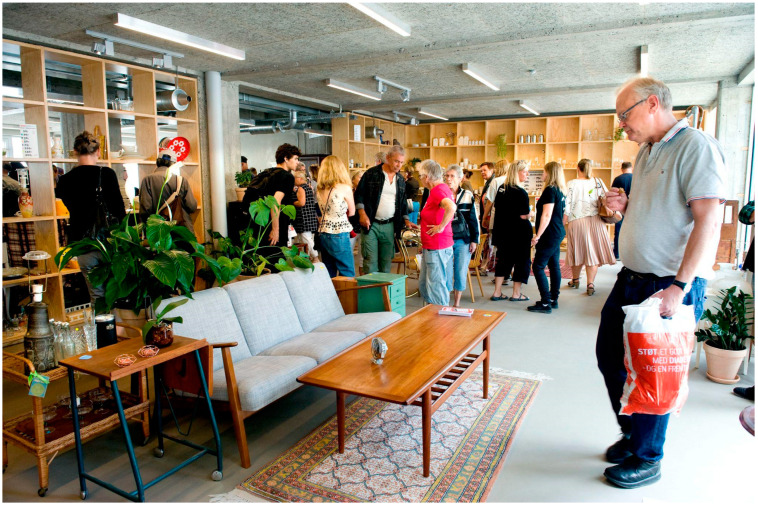
Furniture on the opening day; photo by L. Tuxen for Realdania.

**Scheme 6:**
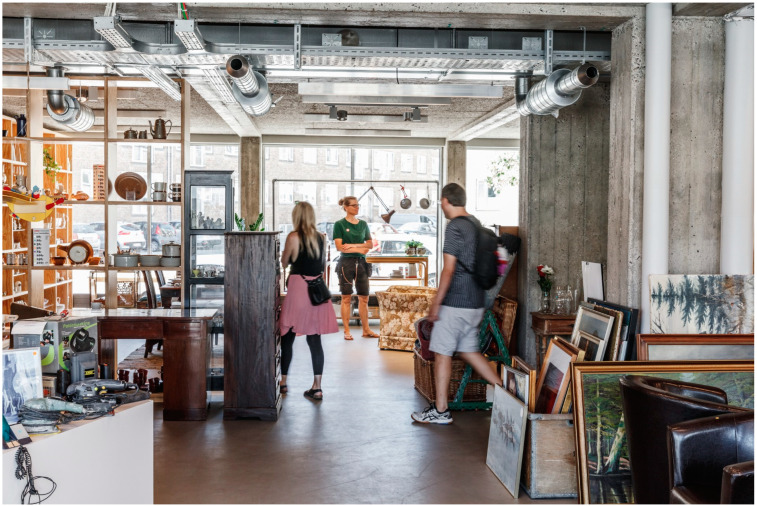
Furniture, Photo: R. Hjortshøj for Kofoeds Skole.

As opposed to Clothes, there is no clear distinction between the workspace (School) and the shop (City) in the Furniture department (see Figure 6). The two zones are one and the same, due to the size of the objects being sold and needing to be stored (various furniture items such as chairs, lounge chairs, coffee tables, and smaller items such as kitchen equipment, etc.). This means that when Furniture receives donations of large items to be sold in the shop such as a bed, a bookcase or table, they are received through the backdoor of the shop and most of the time taken directly to the shop. Smaller items such as plates, cups, or nick-nacks can be stored in a small corridor, but often they are placed on a table in the back end of the shop before a place to exhibit them is found. This creates a more confusing work routine for the students, as there is no clear distinction between workspace and public area, and they always need to consider where to put things to make sure the shop looks inviting to customers (see Scheme 5 and Scheme 6).

Another difference between Furniture and Clothes is that the Base area in Furniture is placed in an adjacent room, with no visual link between the Base area and the rest of the Furniture area, making it two separate settings, rather than conjoint subsettings. If a student needs to take a break in Furniture, he/she needs to pass through two doors and a small, dark hallway to get to the room (see Figure 6). This results in a lack of visual and auditory connection with what goes on in the workroom, including the arrival of new donations or customers. Despite having a Base, the students report finding it more difficult to sneak next door and take a break. The combination of the small hallway and two doors, as well as the sense of being excluded, creates physical and mental barriers resulting in the Base rarely being used. Here, it seems that students lack the *in-between* or *both-and* space that was more apparent in the Clothes section; in other words, the architectural layout of Furniture does not, to the same extent, afford or enable a liminal participation. Consequently, the physical layout renders participation in Furniture more demanding, and in general it is described by both staff and students how you need to be ‘tough’, and able to work alone, to work here:

You must be able to handle something to be here. It’s not the most vulnerable students who should be here. In that case, they can go to the Production Workshop [a sheltered workshop at KS], where there’s only one task to focus on and it’s quieter. So, there’s different workshops depending on how you feel.

(Conversation with Kim, a student)

Furthermore, the architectural layout of Furniture means that students often work alone, either in the workspace or the shop, as there is no centrally placed table or area where they can sort through the donations. Instead, a lot of their work is carried out along the edges of the room, sorting through items and ordering the shelves or exhibitions, which makes it difficult to have conversations with one another. And so, the Furniture section as a behavior setting seems to work more as a collection of subsettings affording different actions that are primarily carried out alone. This may work well for those students who prefer solitude; however, a majority of students use (and depend on) the school for socializing. Thus, being unable to converse naturally in a shared workspace makes them much more dependent on one-to-one interactions with the staff. This in turn reinforces the sense of needing more formalized support and may not in the same way contribute to the development or expansion of action possibilities. As one student explained:

“*If you have a confusing private life, and you arrive in the morning and don’t know what your tasks are, then that’s a bad cocktail.*”

Similarly, the teacher in Furniture explained:

You must be able to cope with me not being next to you. At Clothes, they’re good at going next door and having a cup of coffee. It’s not like that here. Things are constantly arriving, that we need to get inside. So, if you need coffee, this is not the place. People often stand by themselves during the day. So, it can be a difficult place to be if you come for the social aspect.

In Furniture, the complete merge of workspace (School) and shop (City), and lack of an easily accessible Base, risks enhancing or reinforcing a distinction between perceiving yourself as a student (in need of support) or a working person (who can more independently carry out work functions). For students who work well independently and who are more robust, Furniture on the other hand provides many interesting tasks and challenges. The students take pride in figuring out the value of a donated item, showcasing the products in an intriguing way and making sure the shop looks inviting. A student highlights how he loves being at the Furniture section as it has provided him with a sense of having a professional identity. For students who are more vulnerable and have (more) days where they are feeling anxious, however, Furniture risks doing the exact opposite. The constant influx of donated furniture means that the place easily looks messy or becomes unmanageable, which may cause students to feel responsible, in the sense of not having done their job properly. Here a liminal hotspot may arise in the sense that students may feel left alone and stressed out in a setting that is all about support, therefore it may be difficult to ask for help or understand why feelings of anxiety arise in an otherwise very supportive setting.

##### Clothes versus Furniture 

The comparison of Clothes and Furniture demonstrates how the architectural layout may contribute to different experiences of action possibilities for the students: (1) by working with conjoint subsettings in the Clothes section that allow students to move freely and flexibly according to their needs, without leaving the Clothes practice; and (2) in the Furniture section, the separation of various functions (School and Base) in different behavior settings seem to counter not only students’ sense of control and agency, but also the possibility to experiment more freely with different modes of participation without leaving the behavior setting and hence the feeling of being socially connected.

Here it becomes evident that even though the general refurbishment of KS seems to work well, the different behavior settings seem to support students’ wellbeing or growing sense of participation, agency, or relevant possibilities for action differently. From our participant observations in Clothes and Furniture, we see how the architectural layout and the way in which the settings invite activities and participation in different ways have an impact on students’ experience of feeling cared for, which arguably seem to contribute with something constructive in relation to their self-understanding as capable subjects. Furthermore, the layout of the settings influences how staff may support students’ participation and individual alternations between taking part in work tasks and withdrawing. In Clothes, the visual coherence of the different subsettings means that staff may have an eye on all participants in the setting, even when someone withdraws to the Base area. This makes it easier to follow up on students and to have an idea of different students’ needs (also procedurally and non-verbally). Being able to collaborate on work tasks—even with different modes of participation—fosters a sense of coherence and a community of practice [41,42] where focus becomes the shared task, in this case processing clothes for recycling and sale. As also emphasized by Minken [41], it is often easier to have conversations about the difficult or challenging aspects of life, if these are anchored in our shared practice of *doing something together*. And here, the Clothes department as a behavior setting much more directly affords collaboration and shared engagement in work tasks compared with Furniture, where one is most often left to work alone or without visual connection with others due to the architectural layout. Not only do staff members often struggle to maintain a sense of students’ well-being in Furniture, but students also find it difficult to connect with other students, as the room to a lesser degree lends itself to shoulder-to-shoulder work tasks.

#### 5.2.2. Exploring the Relation between School and Public Spaces (School-City)

Research from 2017 by The Danish Centre for Social Science Research (VIVE) found that over half of the citizens experiencing social vulnerability feel disenfranchised and four out of ten do not feel acknowledged or appreciated in their everyday lives [42]. The Director of the Kofoed School, Robert Olsen, similarly describes how “Homeless people, mentally ill people, and other vulnerable groups take on a societal role as part of a subculture that is defined by others and that implies societal exclusion” [43] (p. 78, our translation). These statements point at the importance of creating spaces, where people who experience social vulnerability or societal exclusion can gain a sense of inclusion, and experience acknowledgement as fellow citizens, rather than marginalized outsiders.

The new behavior settings instated by the Base-School-City principle not only change the ways in which the different workshops/workspaces function. The new openness towards the public, brought about by the City-aspect, also enables a shift in the narrative of KS as an institutional setting, which in itself may contribute to overcome the marginalization experienced by the students. Like many similar social institutions, KS has long endured the reputation as a notorious place for drunks and drug addicts, meaning that attending the school has not in itself contributed to overcome issues of marginalization or societal exclusion, but has perhaps even served to reinforce them. And so, it is not only within the workshops that the renovation has created liminal spaces of participation. In the following section we will highlight some of the ways in which the social economic shops and the relation between city and school enable shifts in narratives and in student’s self-understandings by creating a liminal space of participation where one may at the same time participate from a marginalized position as well as experience a more acknowledged societal position.

##### A Liminal Space of Possibilities

**Scheme 7:**
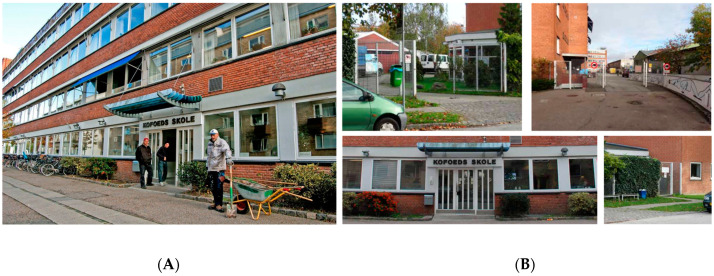
The front entrance before the renovation (**A**), and Before the renovation: fences and gates closed off the school for the public (**B**). Photos: Cobe Arkitekter.

**Scheme 8:**
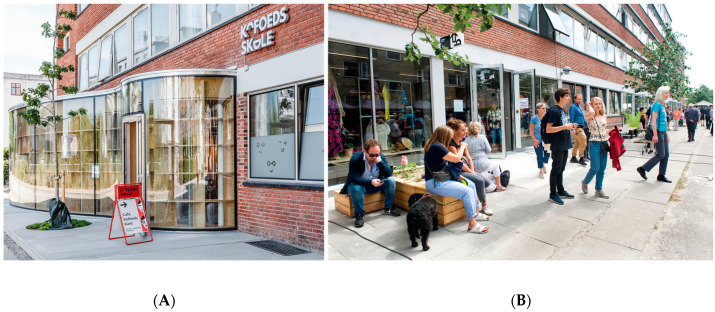
The café entrance after the renovation (**A**), and The open doors from one of the shops (Clothes) (**B**). After the renovation: shops and the café open up the facade for the neighbourhood. Photos: R. Hjorthøj for Kofoeds Skole and Leif Tuxen for Realdania.

At KS, shops and the cafe are open to the public (see Scheme 7 and Scheme 8 for before and after). Here students and outside guests are customers on equal terms: side by side, they browse through the same shelves or sit next to one another sipping coffee. Shopping, choosing your own furniture or clothes, or using a café, is for many of the students at KS a rare luxury that is usually out of reach due to their financial situation (being on social benefits). However, when working in the workshop, the students earn a small salary paid via coupons which they can use in the shops or café—to pay for a new shirt, a considerable number of cappuccinos, or a new pair of shoes or woolen socks (The students are not allowed to receive a real salary from the school due to regulations for being on unemployment benefits). Previously, the school offered opportunities for students to receive things such as clothes through the workshop Clothes, however only once a month and as a charity. At this point in time, Clothes was not an actual shop but a place where students could—free of charge—pick out clothing items that they needed, and solely for themselves. By creating the shops, students are now able to purchase what they want, rather than what they need. As a member of the staff explained:

The fact that the shops may contribute to remove the clientization that often arose when students could only come by once a month and in addition had to report what they took to the staff-person in charge… Now they purchase stuff like anywhere else, and it also adds positively that they have to pay like everyone else.

(conversation with shop manager (staff))

In our conversations with students, many highlighted the meaning of being able to shop—and pick out things themselves—as one of the main benefits of the transformation at KS. Equally important, however, is the possibility to host and invite friends or family inside to a place that feels like home and that fosters pride and allows them to produce, to purchase and give gifts, or to be able to pay for a coffee for someone else. Together, these new possibilities for action that are enabled and afforded at KS contribute to a sense of independence, normalization, and societal inclusion. With reference to Davidson [18] as well Topor, Larsen and Bøe [19], this constitutes an important foundation for recovery from mental illness or psychosocial issues. Equally, as Warming and Fahnøe have pointed out, independence is “a dominant citizenship norm”, and thus being able to provide for yourself or others ties in with a sense of citizenship [22]. 

While these effects are partly caused by the pedagogical strategy, e.g., by paying the students through coupons for the shops, the physical surroundings also play an important part. One student noted that rather than looking like a storage room, Clothes now appears inviting and structured— “It’s almost like a high-end shop in the center of the city!” She appreciates that her activities at KS are now visible to outsiders, inviting them to revise their opinions about the school and its students. Like many of the other students, she experienced how previously people were prejudiced about the school, “but now they can see who we are and that we’re not that bad after all”. She explains that she used to be homeless, and when she first came to the school she begged for clothes. Now she is involved in the running the shop, sorting out the clothes, and this makes her proud. She takes pride in the shop looking neat, and that only the best and most attractive clothes are displayed in the clothes racks. The same student shares an episode where her mother and sister visited the shop. Their knowledge of the school was limited and linked to the negative rumors. However, when they entered the shops, they were really impressed with the look of the place and the products sold. Now they recommend the shops to others. In this sense the new shops and the interesting architecture bridges the prejudice that many students experienced, not only from strangers and the greater public but also within close, social relations such as family. The school and shops enable students to show friends and family, whom they might have broken or difficult relationships with, a side of themselves which is resourceful. 

Besides students’ friends and family, customers in general regularly compliment the appearance of the shops and the products being sold here. A couple of college students told us that KS has the best second-hand shops in town as they roamed through the clothes racks, and a neighbor described it as a ‘hidden gem’. Such compliments serve as an important recognition of the work the students do at the school, which is a rare experience for many. 

The new behavior settings with shops, compared to sheltered workshops only, thus change not only the narrative about the school, but furthermore allow students to experience positive feedback on their products more directly. This in turn feeds into new narratives about themselves as being capable. The acknowledgement that students experience by being able to see their products sold, or by hearing compliments about the workshops, is a direct effect of the architectural layout, where School is perforated by City, inviting the public into the school. Rather than serving as a shield or demarcation line from the public, the school’s outer facade (of glass, see Scheme 8) now serves as an inviting and permeable borderline between the school and the public space, allowing for a plurality of interactions and encounters. Not only does it invite outsiders *into* the school premises, but it also allows students at KS to have positive exchanges with people they would not otherwise engage with or feel seen by. As many of the students’ statements reveal, these experiences of recognition and societal validation make them feel better about themselves, which in turn increases their motivation to take part in new activities or work tasks, contributing to an expanded scope of possibilities for participation and starting anew. In our opinion, this is a potent way for social work to move forward, as students’ increased sense of inclusion and action potential indeed may facilitate recovery (here also in the sense of regaining social meaningfulness and a sense of community) and societal participation, outside the school premises.

The liminal space created by the School-City integration—being *both-and*—contributes to overcoming the marginalization often experienced by students in other settings. Many students report feeling alienated and ‘wrong’ when entering commercial cafés or shops, but at KS it is the other way around. Here, as one staff member explained, the ‘normal’ becomes the guest, who is invited into the domain of ‘the special’:

If one of our students goes to a café, then you’re kinda walking as the only person into ‘the normal’, right. But here, the ‘normal’ enters the special, so to speak. And there is a big difference—I mean besides the economy in it—it is expensive everywhere else. But there is also a big difference in how exposed you feel, by entering a room where you basically do not feel like you belong. And here, there’s no doubt that it’s **their** room. **They** belong here, and then ‘the normal’ may enter their space, and I think that’s the big difference.

(Emphasis as present in conversation)

And so, by explicitly addressing the School-City divide in the architectural layout, KS has succeeded in creating what students and staff alike describe as a safe environment for participation; both in the shops and in the cafe, providing students with a setting in which to experiment with their ‘degree’ of participation and where they may simultaneously acquire new and more positively defined experiences with ‘the outside world’.

## 6. Discussion: Enabling Spaces as Liminal Spaces of Participation

Considering (institutional) efforts directed at people in marginalized life positions or suffering various forms of psychosocial issues rarely includes the physical surroundings. Moreover, such efforts tend to emphasize pedagogical or therapeutic approaches as a supplement to social work interventions to secure basic needs (e.g., a stable home base).

However, as the extensive studies by Barker, Wright, and colleagues [30,31,32,33,44] suggest, the strongest predictor of human behavior is the behavior setting, pointing to the person-environment reciprocity as the most foundational unit of analysis. In line with this perspective, it is not only relevant but crucial to include the concrete architectural and material surroundings when designing institutional settings for social work. In Denmark, most social institutions such as day centers, community centers, or institutional efforts aimed at building work competences such as Kofoed’s School are often *assigned* a building (from the municipality) on short notice, which leaves little time or opportunity to consider (not to mention change) the design or functionality. Furthermore, social institutions are often driven by voluntary work and donations, which similarly makes it difficult to prioritize and invest in the physical setting [45] (p. 14). In effect, the physical settings are rarely considered something to be altered—they tend to become a given—and often, resources are not allocated to consider the more fundamental design or furnishings to support either the physical wellbeing or the needs of the users of the place, or the social work that is to be carried out. 

The Universal Design and Inclusive Architecture approaches make it possible to understand the built environment as a resource that can enable both a higher level of parti-cipation for the individual as well as support the social work taking place in an institutional setting. Moreover, this understanding of places, as something that can contain enabling resources and be designed to become more inclusive, ties in with the notion of social sustainability, as it addresses disparities in access and social inequities in access to social services and participation [46]. And, as Duff highlights, the spaces we inhabit are not static entities but constantly changing and dynamic, depending on *who* and *what* is present in the situation, causing places to have “diverse effects, sometimes enabling, sometimes indifferent and sometimes detrimental or even harmful” [47] (p. 155). When programming and designing the physical settings of social institutions it is therefore crucial to understand the various needs from different user groups—both end-users and staff. Our analy-sis of KS highlights that when designing for people who are vulnerable and (often) more sensitive (e.g., to sensory stimuli), the small details, such as material, sightlines, or placements of furniture—as well as the overall layout of the space—contribute to the creation of an inclusive and enabling spatial setting. 

In this article, we have explored how in the redesign of Kofoed’s School in Copenhagen, Denmark, the architectural layout has been successfully included in the consideration of the invitational character of the different locations on the school’s premises. By carefully redesigning the various behavior settings of the shops and the cafe area, the possibilities for action as well as the experience of ownership and citizenship for the students have been facilitated, supported, and developed. In other words, the concrete surroundings now afford (acc. Gibson [26]) different ways of participating for students, or perhaps we should rather say that a multiplicity of behaviors are now afforded that may allow room for variation according to students’ needs. Before the redesign, the same workshops contained sharper demarcation lines between participation and non-participation, or in- and exclusion in the various activities. Now students can more fluidly, at least in Clothes, alter their way of participating without feeling excluded, just as the demarcation lines between inclusion/exclusion is challenged with the new School-City relationship, as we saw in the analysis. Here, it seems, the ambiguous and paradoxical nature of liminality has successfully been used to create *spaces of possibility* for people in otherwise marginalized or ‘locked’ life positions. Returning to the notion of the liminal hotspot, this is generally considered an experience of suspended “limbo of an in-between phase of transition” [37] (p. 142), where uncertainty, ambiguity, and tension is noticeable. We fully recognize how this may often be the case, just as it may also be at KS. However, when spending time at KS, taking part in the everyday life at the school, it becomes apparent that students do not primarily experience their participation here as tense, uncertain, and ambiguous in a negative sense. Rather, it is quite the opposite: because KS has managed to create a plurality of settings that, in different ways, play with liminality as a constitutive premise, allowing students to be in *both/and* positions in relation to their participation; students are able to use the ambiguousness and uncertainty as a space of possibilities that they did not experience before. This in general seems to have a positive impact on students’ experiences of wellbeing, socially as well as in relation to their mental health. We have not conducted statistical evaluations on this matter specifically, but from our empirical material, these are the impressions conveyed by both students and staff.

It would be too simple to claim that the school marks the liminal phase between two clearly marked life phases; this would install a normativity of all students moving, in a linear fashion, towards ‘full’ societal participation in the shape of employment (outside KS) and (system) independence. This is far from the case. Instead, it is much more potent to reflect upon the ways in which KS succeeds with co-creating spaces of possibility for their students by enabling a multitude of forms of participation, with no clear direction or form to master. The ontological indeterminacy that lingers in the liminal hotspot, according to Greco and Stenner [37], reminds us that many of the students are still struggling to find their form. Enabling this space for students to experiment and experience themselves in new roles or positions is in our opinion a meaningful and supportive measure.

At the same time, however, not all students experience the new design as supportive, and feelings of societal exclusion are not necessarily overcome simply by ensuring societal exposure or visibility. Thus, to some, the fluid and more permeable borders between School and City—between shielded and exposed—may be difficult to handle and may indeed place them in a liminal hotspot in a negative sense, where the tension becomes too intense and where they have difficulties in finding relevant action possibilities. As a consequence, not showing up at KS may feel like social exclusion while being there may leave them feeling too exposed. This makes it an ongoing challenge of co-creating legitimate ‘middle-grounds’ or ‘spaces between’ participation/non-participation and exposure/shielding that enable exploration, experimentation, and (peripheral) participation (in line with Lave and Wenger’s notion of situated learning [48]), while ensuring a sense of security.

As we saw in the analysis, the different workshops come with different architectural layouts, just as there are workshops that do not allow public access. With this plurality of settings, KS as an institutional setting may mediate the border-work that students have to do as part of their development. If tension is too high when participating in Furniture, one may change to Clothes, or to a non-public sheltered workshop. This is where the staff comes in; as mediators of individual needs, motives, and competences—supporting students in their own personal trajectory, moving in liminal spaces of possibility. When we propose to think along the lines of ‘enabling spaces’, we do so with an intent of bringing awareness to how the architectural layout, the concrete design of the room and its functionalities, may contribute productively to the facilitation of institutional settings that at the same time are experienced as supportive and open to experimentation. In Table 1, we highlight some of the architectural solutions or remedies that have been implemented at KS to support (1) students’ needs, and (2) the school’s pedagogical and social approach, thereby creating what we propose to label enabling spaces. The table is not exhaustive, nor is it meant as a one-size-fits-all design guide to social work efforts. However, it is our hope that it may serve as inspiration for how one may include the physical layout in the planning of institutional settings within the field of social work.

According to Siren et al., [1]; Ellard [49]; and Roe and McCay [50], a broad variety of research explores the relationship between the built environment and human health, wellbeing, and social relations; examples include evidence-based design and therapeutic environments within the health sector. However, when it comes to designing for people who experience marginalization and social exclusion, there is a knowledge gap [1]. This is partially due to the fact that social institutions are rarely alike; they are specialized in serving a particular segment of this population but are at the same time rarely custom built. Moreover, the institutions are often relatively small in scale. This makes it difficult to produce broad conclusions on which (and how) physical aspects may enhance the respective social work efforts and how it, directly, affects the wellbeing of the users (in a causal manner) [45] (p. 62), [1]. 

Our case analysis of KS furthermore highlights the importance of even the smallest physical details when building for people who are vulnerable or, as this group often are, highly sensitive to their surroundings. The distance, visual connection, type of door between the School- and Base-zones, as well as the decor, play important roles in the way in which students are able to participate and navigate between different positions. But this does not change the importance of continuing to research and evaluate how the physical surroundings at social institutions may contribute to the creation of inclusive and enabling spaces. However, rather than aiming at a one-size-fits-all design manual, we argue for a plurality of rich case examples to demonstrate how enabling spaces may be constructed in various ways. 

## 7. Conclusions

With the renovation KS has, in many respects, succeeded in establishing what we could call a *productive liminal space of possibility* in the sense of providing a steppingstone or a middle ground in a journey towards an increased experience of societal inclusion (or more recognized societal participation) for people in marginalized positions. Hence, they have successfully addressed the challenge or the need for relevant possibilities for participation, or adequate ‘spaces between’ participation and non-participation. It is the possibilities for experimentation, action, and flexibility instated by these liminal positions of being ‘in between’ or ‘both/and’ that we consider *enabling spaces*: institutional spaces that invite you to become anew, at your own pace, while simultaneously presenting new demands and offering support.

In that sense, institutional settings are not neutral spaces, offering similar possibilities irrespective of their specific material and architectural layout. Rather, such spaces may offer a variety of possibilities and affordances for its participants to respond to, engage with, and make use of. Therefore, it is pivotal that the physical surroundings are considered a resource when planning and designing social work efforts, as well as in cases where the target group may be complex in nature. It is of utmost importance that we consider our design of work and learning spaces to be inclusive; also, when inclusive means securing spaces for withdrawal. This may be a non-verbal way of letting students know that feeling vulnerable, over-exposed, or anxious is accepted and acknowledged. Our observations from KS point to these feelings of acceptance and acknowledgement as some of the components that motivate students to engage in new activities and challenge themselves in new ways.

Our case study of KS reminds us that well-being, recovery, and citizenship may be facilitated in numerous ways, and that some of these have to do with the concrete environments in which we engage with one another: student-to-student, staff-to-student, student-to-public. Not only may the new openness at KS be beneficial to students, who are experiencing a wider range of action possibilities, but it may also help minimize the stigma towards people in marginalized positions when the public is invited to take part in new meetings and experiences, e.g., in the shops and the cafe. This way of bridging people that may otherwise live parallel lives may indeed contribute to more inclusive societies.

## Figures and Tables

**Table 1:** Presents an overview of the social challenges or problems that are addressed at KS, along with the concrete architectural solutions, and the social effects.

Social Challenge/Problem	Architectural Solution	Social Effect
Vulnerable life position due to social marginalization, somatic, and/or mental health problems causing the students to experience barriers for participation within the build environment	Conjoint but distinct subsettings allowing for different types of actions (e.g., work, learning, respite)	Allows students to easily move between different settings depending on their mood and mental wellbeing (also without drawing too much attention to themselves or entirely leaving the setting) Functions as a pedagogical tool and learning space for students to acquire and/or develop skills and familiarity in relation to participating in more professional settings.
	Transparency/visual connection between rooms/subsettings	Affords students the possibility for breaks without having to feel excluded. Enables staff to be aware of what is happening in the workspace and, at the same time, see to a student who needs extra attention or support. Allows students to prepare themselves for what awaits them in the next room/subsetting, e.g., to mentally prepare themselves to enter a space with many people, therefore it eases transitions.
	Interior layout and furniture convey visible work positions and tasks	Makes it easier for students to carry out their task and know where to position themselves, even though they may not be familiar with the work. Decreases stress and confusion related to deciphering work tasks
	A central, communal worktable or meeting space	Affords space for collaboration and shared work tasks Provides something concrete and tangible to meet around and facilitates social interaction and collectivity, also in relation to establishing common thirds and communities of practice [48]
Experience of social exclusion and stigma	Introducing social economic shops on the ground floor.	Allows the public to use and see the school as a local asset, thus allowing them to know the school. Allows students and neighbors to use the same spaces, hence facilitating more dynamic meetings. Enables the exhibition and sale of students’ products, thereby giving them a sense of worth and pride. Allows students to shop and invite friends or family for a cup of coffee on equal terms with others (by granting ownership of the school premises).
	Opening up the façade and school towards the public.	Affords a new image of the school and its students. Enables the public to see what is going on and become more familiar with the school activities. Provides an active ground floor and façade with more people in the street, which enforces a sense of safety.

## References

[1] Siren A., Grønfeldt S.T., Andreasenog A.G., Bukhave F.S. (2019). Sociale Mursten: En Forskningskortlægning af Fysiske Rammers Betydning i Velfærdsindsatser.

[2] Hendren S. (2020). What Can a Body Do? How We Meet the Built World.

[3] Hamraie A. (2019). Building Access: Universal Design and Politics of Disability.

[4] Papanek V. (1971). Design for the Real World: Human Ecology and Social Change.

[5] Grangaard S. (2019). Universelt Design—et Begreb i Udvikling. https://www.rumsans.dk/artikler/universelt-design-et-begreb-i-udvikling.

[6] (2008). The Center for Universal Design. https://projects.ncsu.edu/ncsu/design/cud/about_ud/about_ud.htm.

[7] Universaldesign.ie Policy and Legislation. https://universaldesign.ie/what-is-universal-design/policy-and-legislation/policy-and-legislation.html.

[8] Edward og Maisel S., Jordana L. (2012). Universal Design: Creating Inclusive Environments.

[9] The EIDD Stockholm Declaration. https://dfaeurope.eu/what-is-dfa/dfa-documents/the-eidd-stockholm-declaration-2004/.

[10] Dansk Standard (2021). Europæisk Standard for Universelt Design Skal Sikre Tilgængelighed for Alle. https://www.ds.dk/da/nyhedsarkiv/2021/11/europaeisk-standard-for-universelt-design-skal-medvirke-til-tilgaengelighed-for-alle.

[11] European Commission (2021). Common EU Standards for a Built Environment Accessible to All. https://europa.eu/new-european-bauhaus/get-inspired/selection-your-contributions/common-eu-standards-built-environment-accessible-all-2021-04-30_en.

[12] Ryhl C., Høyland K. (2018). Inkluderende Arkitektur.

[13] Inclusive Design Toolkit. https://inclusivedesigntoolkit.com/whatis/whatis.html.

[14] Inclusive Design Research Centre Philosophy. https://idrc.ocadu.ca/about/philosophy/.

[15] Grangaard S. (2018). Analyse af Bygherrens Tilgang til tilgængelighed og Universelt Design i Byggeriet.

[16] Realdania. https://realdania.dk/publikationer/faglige-publikationer/socialemursten.

[17] The A.P. Møller Foundation. https://www.apmollerfonde.dk/socialindsats/programmer/program-3-fokusomraader/.

[18] Davidson L., Oute J., Jørgensen K. (2021). Recovery-bevægelsens historiske og konceptuelle rødder. Recovery Orienterede Praksisser—i Velfærdsinstitutioner og Civilsamfund.

[19] Topor A., Larsen I.B., Bøe T.D. (2020). Återhämtning—Från Personlig Reformering Till Social Förändring. https://madinsweden.org/2020/04/aterhamtning-fran-personlig-reformering-till-social-forandring/.

[20] Delanty G. (2003). Citizenship as a learning proces: Disciplinary citizenship versus cultural citizenship. Int. Lifelong Educ..

[21] Lister R. (2007). Inclusive Citizenship: Realising the Potential. Citizsh. Stud..

[22] Warming H., Fahnøe K., Warming H., Fahnøe K. (2017). Social Work and Lived Citizenship. Lived Citizenship on the Edge of Society.

[23] Om os. https://menneskermedmere.dk/omos/.

[24] carlberg|christensen and COBE (2015). Kofoeds Skole i Fremtiden—Visionsplan for Kofoeds Skole Som Driver for Åbenhed, Bæredygtighed og Socialøkonomiske Aktiviteter i Holmbladsgadekvarteret. https://realdania.dk/-/media/realdaniadk/publikationer/kofoeds-skole/booklet_kofoeds-skole_low.pdf.

[25] Kofoeds Skole (2019). Kofoeds Skole i Fremtiden—Statusrapport. https://menneskermedmere.dk/wp-content/uploads/2019/05/Kofoeds-Skole-statusrapport-2019-web.pdf.

[26] Gibson J.J. (1986). The Ecological Approach to Visual Perception.

[27] Heft H. (1988). Affordances of children’s environments: A functional approach to environmental description. Child. Environ. Q..

[28] Barker R., Wright H. (1971). Midwest and Its Children: The Psychological Ecology of an American Town.

[29] Wright H.F., Barker R.G., Nall J., Schoggen P. (1951). Toward a psychological ecology of the classroom. J. Educ. Res..

[30] Pedersen S. (2021). Developmental Dynamics and Transitions in High School.

[31] Gump P.V. (1965). Big Schools … Small Schools. Paper Published as Part of the Chronicle Guidance Professional Service.

[32] Wicker A.W. (1968). Undermanning, performances, and students’ subjective experiences in behavior settings of large and small high schools. J. Personal. Soc. Psychol..

[33] Hedegaard M. (2012). Analyzing children’s learning and development in everyday settings from a cultural-historical wholeness approach. Mind Cult. Act..

[34] Turner V., Lessa W.A., Vogt E.Z. (1979). Betwixt and Between: The Liminal Period in Rites de Passage. Reader in Comparative Religion.

[35] Turner V. (1969). The Ritual Process: Structure and Anti-Structure.

[36] Greco M., Stenner P. (2017). From paradox to pattern shift: Conceptualising liminal hotspots and their affective dynamics. Theory Psychol..

[37] Stenner P., Greco M., Motzkau J.F. (2017). Introduction to the Special Issue on Liminal Hotspots. Theory Psychol..

[38] Kristiansen S., Krogstrup H.K. (2015). Deltagende Observation.

[39] Musante K., DeWalt B.R. (2010). Participant Observation: A Guide for Fieldworkers.

[40] Pedersen M., Pedersen M., Klitmøller J., Nielsen K. (2012). Triangulær validering. Samspillet mellem deltagerobservationer og kvalitative interview. Deltagerobservation—En Metode til Undersøgelse af Psykologiske Fænomener.

[41] Minken A. (2002). Alvorlig Moro: Idé og Virksomhet ved Motorsportstiltaket 2&4.

[42] Jørgensen T. (2017). Fællesskabsmålingen—En Undersøgelse af Livsvilkår og Social Eksklusion i Danmark.

[43] Christensen E., Olsen R. (2018). Kofoeds Skoles ABC.

[44] Pedersen S., Bang J. (2016). Historicizing affordance theory: A rendezvous between ecological psychology and cultural-historical activity theory. Theory Psychol..

[45] Carlberg N., Lind H. (2017). Sociale Mursten. Den A.P..

[46] Dempsey N., Power S., Bramley G., Brown C. (2009). The Social Dimension of Sustainable Development: Defining Urban Social Sustainability. Sustain. Dev..

[47] Duff C. (2011). Networks, resources and agencies: On the character and production of enabling places. J. Health Place.

[48] Lave J., Wenger E. (1991). Situated Learning: Legitimate Peripheral Participation.

[49] Ellard E. (2015). Places of the Heart: The Psychogeography of Everyday Life.

[50] Roe J., McCay L. (2021). Restorative Cities—Urban Design for Mental Health and Wellbeing.

